# Assessing Distinct Cognitive Workload Levels Associated with Unambiguous and Ambiguous Pronoun Resolutions in Human–Machine Interactions

**DOI:** 10.3390/brainsci12030369

**Published:** 2022-03-11

**Authors:** Mengyuan Zhao, Zhangyifan Ji, Jing Zhang, Yiwen Zhu, Chunhua Ye, Guangying Wang, Zhong Yin

**Affiliations:** 1 College of Foreign Languages, University of Shanghai for Science and Technology, Shanghai 200093, China; mengyuan.zhao@usst.edu.cn; 2 Shanghai Key Laboratory of Modern Optical System, Engineering Research Center of Optical Instrument and System, Ministry of Education, University of Shanghai for Science and Technology, Shanghai 200093, China; 202440428@st.usst.edu.cn (Z.J.); 202440470@st.usst.edu.cn (J.Z.); 202440468@st.usst.edu.cn (Y.Z.); 192550424@st.usst.edu.cn (C.Y.); 192550434@st.usst.edu.cn (G.W.); 3 School of Optical-Electrical and Computer Engineering, University of Shanghai for Science and Technology, Shanghai 200093, China

**Keywords:** electroencephalograph, pronoun resolution, cognitive workload, machine learning, principal component analysis

## Abstract

Pronoun resolution plays an important role in language comprehension. However, little is known about its recruited cognitive mechanisms. Our investigation aims to explore the cognitive mechanisms underlying various types of pronoun resolution in Chinese using an electroencephalograph (EEG). We used three convolutional neural networks (CNNs)—LeNeT-5, GoogleNet, and EffifcientNet—to discover high-level feature abstractions of the EEG spatial topologies. The output of the three models was then fused using different scales by principal component analysis (PCA) to achieve cognitive workload classification. Overall, the workload classification rate by fusing three deep networks can be achieved at 55–63% in a participant-specific manner. We provide evidence that both the behavioral indicator of reaction time and the neural indicator of cognitive workload collected during pronoun resolution vary depending on the type of the pronoun. We observed an increase in reaction time accompanied by a decrease of the theta power while participants were processing ambiguous pronoun resolution compared to unambiguous controls. We propose that ambiguous pronoun resolution involves a more time-consuming yet more flexible cognitive mechanism, consistent with the predictions of the decision-making framework from an influential pragmatic tradition. Our results extend previous research that the cognitive states of resolving ambiguous and unambiguous pronouns are differentiated, indicating that cognitive workload evaluated using the method of machine learning for analysis of EEG signals acts as a complementary indicator for studying pronoun resolution and serves as an important inspiration for human–machine interaction.

## 1. Introduction

In natural language, a referential pronoun is often used to denote an previously mentioned individual [1]. Pragmatically, a significant role of pronouns is to connect new information to what has already been presented in the context [2]. Pronoun resolution is thus a fundamental process in language comprehension [3]. Although it is argued that the readers determine pronoun referents mainly based on the gender of the pronoun [4], this task is complicated by the fact that pronouns may present referential ambiguity, where the gender information is insufficient for referent identification. The resolution of ambiguous pronouns, which pose a challenge in language comprehension, has aroused wide interest in the transdisciplinary research field of linguistics, psychology, neuroscience, and machine learning. For instance, the Google AI team has recently presented and released a gender-balanced corpus of 8908 ambiguous pronoun–noun pairs based on real-world text [5].

In the field of human–machine interaction, understanding the cognitive states of language comprehension becomes increasingly important, as it could be applied to improve the working efficiency of artificial intelligence. Discovering the cognitive mechanism underlying pronoun resolution is a critical component of understanding human language comprehension. Approaches for characterizing the cognitive procedures of pronoun resolution vary by level of analysis. At the behavioral level, psycholinguists normally study pronoun resolution via analysis of behavioral data, such as response accuracy and reaction time collected from designed questionnaires [6,7]. A group of psycholinguistic studies investigated how a reader identifies the referent of a pronoun. In the cases of unambiguous pronoun resolution, gender information was mainly used to determine the referent of a pronoun [4]. In comparison, ambiguous pronoun resolution required more information from linguistic cues, such as verb-bias, focus, and implicit causality [8,9]. A limitation of behavioral analysis is that its reliability largely depends on the experimental design. For instance, when certain task circumstances require a human operator to perform manual operations without expressions and interruptions, data from rating questionnaires and external behaviors is difficult to collect [10,11].

At the neural level, cognitive neuroscientists have attempted to reveal the neural correlates underlying the cognitive procedure of pronoun resolution. Neural measures provide complementary information to discover the cognitive function of language. Neurophysiological signals have attracted much attention, since they can be decoded to investigate the inner cognitive state [12,13,14]. A handful of studies based on fMRI tried to identify the neuroanatomic mechanism of pronoun resolution as related to a decision-making process. In a review work, Chen argued that the reward and cost evaluation in the process of decision making was correlated with an activation in the forebrain structures [15]. Nieuwland et al. studied the brain activity during pronoun resolution in Dutch and identified the recruitment of decision-making-related neural correlates (including media frontal, right superior, medial parietal, and bilateral inferior parietal cortex) while resolving ambiguous pronouns compared to unambiguous cases, suggesting ambiguous pronoun processing involves a cognitive process of decision making [16]. McMillan et al. studied pronoun resolution in English and identified the involvement of brain areas related to probabilistic evaluation (dorsolateral prefrontal cortex) and risk evaluation (orbital frontal cortex) during ambiguous pronoun resolution [17]. These empirical results are consistent with an influential trend of pragmatic tradition, namely Gricean theory for pragmatic reasoning [18]. According to a game-theoretic framework of Gricean tradition [19,20,21,22,23], ambiguous pronoun resolution will trigger a cognitive mechanism of decision making. It is argued that readers resolve an ambiguous pronoun following two cognitive steps [24]: first, they strategically select the referent of the pronoun in a probabilistic way, maximizing the possibility of correctly understanding the pronoun sentence; second, they evaluate the likelihood of selecting the incorrect referent of the pronoun, minimizing the risk of misinterpreting the sentence. On the other hand, a small number of ERP studies have provided neural information on pronoun resolution. Ledoux et al. suggested that the processing of pronoun resolution occurs between 250 and 500 milliseconds [25]. According to a review work of Nieuwland and Van Berkum, the processing of ambiguous pronoun resolution will induce a continuous frontal negative-going ERP [26]. Daltrozzo et al. reported different ERP effects, comparing automatic with controlled processes of speech perception [27].

Although the above research was conducted on the neural measurement of reference resolution, not much is known about the essential differences in cognitive workload recruited to support different tasks of unambiguous pronoun resolution and ambiguous pronoun resolution in Chinese. Unlike English, Chinese is a tonal language and thus has suprasegmental features such as pitch changes, resulting in a great number of homophones in the vocabulary [28]. Based on a recent fMRI study, Ge et al. reported the differences in neural correlates underlying speech comprehension between English and Chinese: the latter involves additional activation of the right hemisphere, which has long been considered as largely unrelated to language processing [29]. It is worthwhile mentioning that the electroencephalogram (EEG) with portable sensors can be conveniently implemented in a passive brain–computer interfacing system to measure human cognitive workload [30,31]. Compared to fMRI and ERP, EEG signals provide continuous observation of brain activities through cortical networks at a sampling frequency greater than 1000 Hz [32], with millisecond-range temporal resolution [33]. Thus, EEG data are commonly used to study various human cognitive functions, such as emotion [34,35], language processing [36,37], and memory [38,39]. A challenge for studying the cognitive workload correlated to pronoun resolution based on EEG data is to find approaches to decoding EEG signals across different participants and dimensionalities.

At the level of algorithm, the methods of machine learning and deep learning have received much attention. Ladekar et al. used energy, band entropy, non-linear energy, and signal length to extract features and used a K-nearest neighbor (KNN) classifier to identify cognitive workload [40]. Maldonado et al. computed the statistics (i.e., mean, median, and variance) of each EEG signal, extracted the EEG feature of entropy by discrete wavelet transform (DWT), and employed the support vector machine (SVM) as the approach of feature selection [41]. Based on the features obtained by wavelet transform decomposition of EEG signals and by ensemble subspace K-nearest Neighbor (ESKNN), Gupta and Manthalkar obtained a classification accuracy of 86.12% [42]. Bhattacharyya et al. extracted the temporal and spectral entropies from the multichannel EEG signal. These features were smoothed and applied to the sparse autoencoder random forest (ARF) classifier [43]. Based on a wavelet transformation, Zhang and Wang added time and frequency dimensions to EEG topographic maps and proposed a concatenated structure of deep recurrent and 3D convolutional neural networks (R3DCNN) to identify cognitive workload levels [44]. In recent studies, Zhang et al. proposed the two-stream neural networks to fuse the extracted EEG features from two relevant domains [45]. The feature embedding was generated by combining spectral and temporal information from the spatial distribution of the EEG power. Almogbel et al. applied a 10-layer convolutional neural network to identify a two-level workload under a simulated driving task [46]. The classification accuracy was achieved at 86.5% when four-channel EEG with fast Fourier transformation was applied. Fan et al. evaluated the affective state and workload of adolescents with autism spectrum disorder using wireless EEG signals [47]. By using the leave-one-subject-out cross-validation paradigm, the classification accuracy of the workload was 86%. Accuracies of enjoyment and frustration were 90% and 88%, respectively. Blanco et al. used dry EEG sensors to quantify the workload of flight pilots, and the median of the classification was 90.17% [48]. They found that the feature combination and the classifiers should be personalized to fit the distribution of each participant. Lim and Sourina applied transfer component analysis to identify workload across two independent databases with 48 subjects and 18 subjects, respectively [49]. The average classification accuracy was 30% for four-class classification. Wang et al. applied a wireless EEG system to evaluate human workload under three conditions [50]. Based on a proximal support vector machine, the classification rate was higher than 80%. The above works indicate the effectiveness of using EEG to evaluate cognitive workload under a variety of cognitive tasks.

Our investigation had the goal of studying the underlying cognitive mechanisms of pronoun resolution. Although machine learning and deep learning applied to the analysis of the big EEG data are useful for investigating the underlying neural correlates of pronoun resolution, how EEG signals reveal the cognitive states of pronoun resolution remains largely uncharacterized. The current study aims to explore the cognitive mechanisms of pronoun resolution for two types of pronouns: unambiguous and ambiguous pronouns. We hypothesize that the resolution of unambiguous and ambiguous pronouns involves different cognitive mechanisms. More specifically, we hypothesize that ambiguous pronoun resolution is associated with more than one adequate answer and thus may induce a decision-making process. This hypothesis is consistent with fMRI evidence, since recruitment of decision-making-related neural correlates has been observed [16,17]. Accordingly, compared to unambiguous conditions, people may spend more time choosing a referent for an ambiguous pronoun, as the answer is undetermined, while people may also feel more flexible, as it is less risky to make a mistake. To test these hypotheses, we examined the cognitive workload recruited in pronoun resolution for two types of pronouns. We used EEG to investigate the neural correlates of pronoun resolution. Behavioral data including reaction time and response selection as well as the EEG signals were collected during the pronoun resolution task. We applied pre-trained deep convolutional neural networks (CNNs) to represent spatial patterns of the EEG power features of multiple frequency bands. In particular, two heterogeneous architectures, GoogLeNet [51] and EfficientNet [52], of the deep neural networks were simultaneously employed to find useful hidden information behind combinations of channels and frequency bands. Extracted high-level EEG representations were then fused as a single feature vector to the shallow learning machines to distinguish binary levels of the cognitive workload. Under such a paradigm, the designed deep network could be pre-trained by the huge size of a natural image training set, and then weights of its last layer could be fine-tuned by a specific EEG database with target cognitive workload labels to ensure the generalization capability.

## 2. Materials and Methods

### 2.1. Participants

As an extension of our previous study [53], seventeen volunteers participated in our pronoun resolution task. All volunteers were undergraduate or graduate students from the USST, male, right-handed, in good health with no history of neurological difficulty, and native speakers of Chinese. All participants gave informed consent under the Declaration of Helsinki and a protocol approved by the Ethical Committee of USST (protocol code 1801002). None of the participants had any experience in performing similar cognitive tasks aiming at avoiding the learning effects.

### 2.2. Behavioral Stimuli

As the behavioral stimuli of our experiments, 200 combinations of noun sentences and pronoun sentences were constructed following three steps. First, we identified 40 Chinese gender-neutral nouns and 40 Chinese gender-biased nouns. The nouns were first chosen manually based on a self-built corpus from articles published in a Chinese magazine named *Duzhe*, which is one of the best-selling magazines in China. We then asked 30 students from the University of Shanghai for Science and Technology (USST) to rate each noun according to the gender of the noun. The rules of gender rating are shown in Table 1. On a five-point scale, the student rates a noun (e.g., *taitai* ‘madam’) one point if he thinks it definitely represents a female, a noun (e.g., *hushi* ‘nurse’) two points for being weakly female, a noun (e.g., *tongshi* ‘colleague’) three points for being gender neutral, a noun (e.g., *fanren* ‘prisoner’) four points for being weakly male, a noun (e.g., *xiaonanhai* ‘little boy’) five points for being definitely male. We then converted the five-point scale to a three-point scale: we replaced all of the two- and four-point responses with two points, indicating that the noun is weakly gender-biased; we replaced all the three-point responses with one point, indicating that the noun is gender neutral. A statistical analysis based on the rating data from thirty students suggested that the identified gender-neutral nouns (*M* = 1.33; *SD* = 0.18) were significantly more neutral than gender-biased nouns (*M* = 2.98; *SD* = 0.06; *t* (78) = 54.25; *p* < 0.0001). The statistical analysis justified our choices of nouns according to gender information.

Second, with these 80 nouns, we constructed 200 noun sentences by pairing two nouns with one verb to create each complete and meaningful noun sentence. We constructed the noun sentences with two different syntactic structures: 100 sentences with the structure of subject-verb-object (S-V-O, for short) and the other 100 sentences with the structure of noun-noun-verb (N1-N2-V, for short). Sentences structured as S-V-O appear in both Chinese and English, while those structured as N1-N2-V appear only in Chinese rather than in English. Third, we paired each noun sentence with a pronoun sentence. Each pronoun sentence consisted of a pronoun in the form of the third person singular (i.e., *ta*, ‘he’ or ‘she’) and an intransitive verb (e.g., *xiao*, ‘smile’). Each pair of sentences formed a separate and meaningful context. For each sentence pair, at least one proper candidate could be identified as the referent of the pronoun, whereas no candidates could be excluded in principle by pragmatic reasoning or by the information of linguistic cues (e.g., verb bias, focus, causality, etc.), except for gender information.

We divided the behavioral stimuli into two types: (i) Type 0, where the referent of the pronoun is undetermined by the gender information alone, namely, the pronoun is ambiguous in the context; (ii) Type 1, where the pronoun unambiguously refers to a noun provided with the gender information. Sentences of different syntactic structures (i.e., S-V-O or N1-N2-V) were distributed into the two types of cases. For instance, “*Nanren* (‘man’) *xiangxin* (‘believe’) *nüer* (‘daughter’) *Ta* (‘he’) *xiao-le* (‘smile ASP’) ‘The man believed the daughter. He smiled.’”, with the structure of S-V-O, belongs to Type 1, while “*Qizi* (‘wife’) *he* (‘and’) *xiaonühai* (‘little girl’) *jianmian* (‘meet’). *Ta* (‘she’) *manzu-le* (‘satisfy ASP’). ‘The wife met the little girl. She satisfied.’”, with the structure of N1-N2-V, belongs to Type 0. To cover all the cases, we considered all combinations of conditions, including the gender of nouns, gender of the pronoun, and the syntactic structure. For each combination of the conditions, the number of trials was 20. Of the 200 trials, 120 trials contained ambiguous pronouns (including 60 trials with S-V-O and 60 trials with N1-N2-V), and 80 trials contained unambiguous pronouns (including 40 trials with S-V-O and 40 trials with N1-N2-V). A description of the behavioral stimuli is shown in Table 2.

### 2.3. Experimental Procedure

The experiment was conducted in a clean, silent room at 26 °C, controlled by an air conditioner. The participant sat comfortably in a chair with a distance of approximately 60 cm to a laptop screen that could provide programmed information of the pronoun resolution task. All experiments were conducted in the evening in order to avoid circadian rhythm.

**Figure 1:**
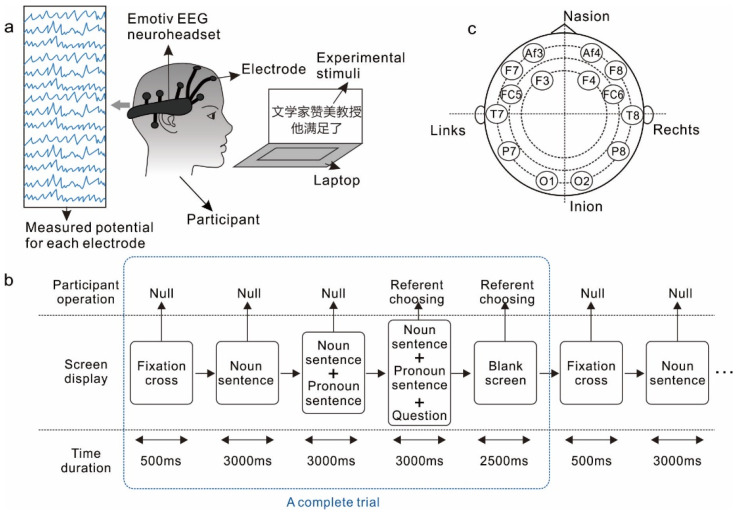
Schematic of the experimental design: (**a**) experimental setting, (**b**) experimental procedure of the pronoun resolution task, (**c**) locations of the 14 electrodes. The Chinese in the picture means: “The litterateur complimented the professor. He satisfied.”

Participants were required to complete the task of identifying the referent of the pronoun in various pairs of Chinese sentences, which were programmed and displayed on a 14 inch laptop computer screen (Dell Windows 10 operation system, Inteli5 CPU 1.6 GHZ, and 12 GRAM configurations). The EEG signals were recorded via an acquisition device, i.e., EMOTIV Epoc+ headset, linked to another laptop computer, and were continuously monitored by an experimenter during the whole procedure. EMOTIV is made up of 16 individual electrodes, of which two are reference potentials, and the other 14 are hydration sensors. Figure 1 illustrates the schematic of the experimental setting, experimental procedure of the pronoun resolution task, and the locations of the 14 electrodes.

Each participant completed 200 experimental trials, and each trial lasted for 12 seconds. Instead of using timing markers, we directly recorded a whole section of the EEG output of each participant, and then segmented the data according to the experimental process to mark the EEG output of each trial. A single experimental trial started by a fixation cross lasting 500 milliseconds, followed by three stimulus events, each lasting 3000 milliseconds. In the first event, the participant was presented with a noun sentence (for instance, *Taitai weixie nüyanyuan*: ‘The madam threatened the actress’) displayed on the computer screen. In the second event, the participant was presented with both the noun sentence and a pronoun sentence (for instance, *Ta shengqi-le*: ‘She was angry’) on the screen. In the third stimulus event, the participant was presented with the noun sentence, the pronoun sentence, and a question that required the participant to choose a referent of the pronoun from the two nouns in the noun sentence. The participant was instructed to choose the noun that appeared on the left (for instance, *taitai*: ‘the madam’ in the previous example) by pressing ‘Z’ on the keyboard or the one on the right (for instance, *nüyanyuan*: ‘the actress’ in the previous example) by press ‘M’. After the question disappeared, a blank screen would last for 2500 milliseconds. From the appearance of the question until the end of the blank screen, the choice of the participant could be recorded. We divided the 200 trials into 5 blocks, and each block contained 40 trials. The participant was allowed a 2 minute break between every two blocks. Before the experiment began, an experimenter introduced details of the number of trials, types of Chinese sentences, and how to answer the designed questions. Prior to the EEG recording session, the participants were allowed to practice ten trials to make sure that they had fully understood the task.

### 2.4. EEG Data Preprocessing and Analysis

**Figure 2:**
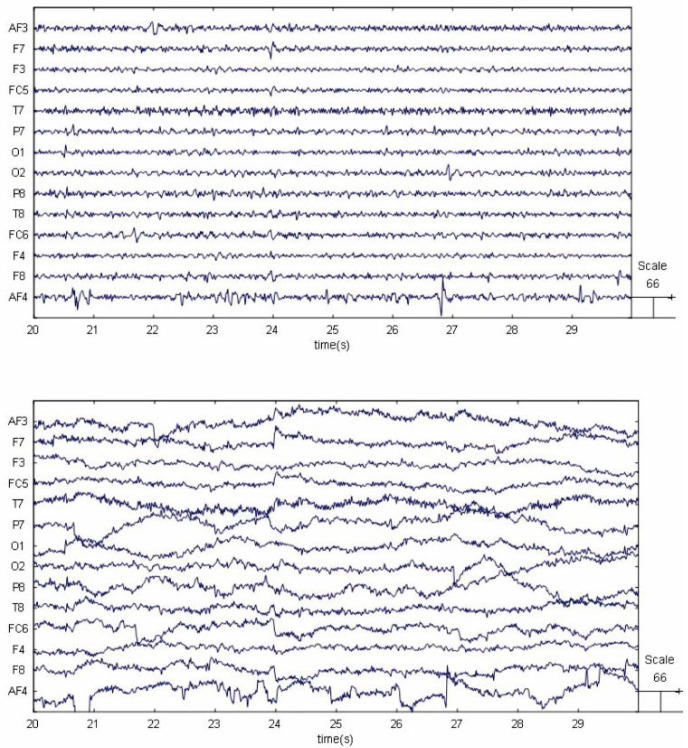
EEG signals before and after bandpass filtering.

When the participants accomplished each trial, we used the EMOTIV Epoc+ (Emotive Systems, San Francisco, USA) EEG headset to record EEG signals of all the participants with a sampling rate of 128 Hz. The employed channel locations labeled with the 10–20 international EEG system are O1, O2, P7, P8, T7, T8, FC5, FC6, F3, F4, F7, F8, AF3, and AF4. EEGlab was used to process the raw data, and the interpolation was carried out to estimate the missing values. Then, the interpolated signals were preprocessed by a linear finite impulse response (FIR) filter. The cut-off frequencies of the passband were set at 4–45 Hz. The comparison of EEG signals before and after FIR passband filtering is shown in Figure 2. Each experimental trial corresponds to a 12 second EEG segment. For each participant, 200 feature vectors corresponding to 200 EEG segments were extracted. For each feature vector, the power of theta (4–8 Hz), alpha (8–13 Hz), and the mean power of beta (14–30 Hz) and gamma (31–40 Hz) were computed by application of the discrete Fourier transform (DFT). Therefore, each EEG segment elicited 42 PSD features (3 features × 14 channels).

Since we plan to use supervised machine learning and deep learning methods to assess workload recognition performance, the target levels (category labels) of the workload for each feature vector should be predetermined. Considering the fact that the sentences in the experiment can be grouped as Type 0 and Type 1 according to the presence or absence of the ambiguous pronouns, the two categories of EEG feature vectors were labeled with 0 and 1, respectively.

Behavioral data such as reaction times and participants’ responses were analyzed during the experiments. In each experimental trial, the specific point in time when a participant chose a referent by pressing ’Z’ or ‘M’ on the keyboard was recorded. The reaction time of the participant in this trial was then computed, given the time information when both the sentence pairs and the question appeared on the computer screen (i.e., the starting point when the participant made the choice). Participants’ reaction times in the cases of ambiguous pronoun resolution were compared to those in unambiguous cases via a two-sample *t*-test. With Type 1 stimuli (the cases of unambiguous pronoun resolution), the participants’ responses were compared to a standard answer, whereby accuracy rates were computed for each participant.

### 2.5. Methods

We present in this section our approach for high-level feature abstractions of the EEG power features to classify pronoun resolution based on three convolutional neural networks (CNNs): LeNeT-5, GoogleNet, and EfficientNet. CNN is one of the most popular deep learning techniques. Initially, CNN is mainly applied to image analysis and shows superb performance on image feature abstraction and classification. It has also been proven that CNN has advantages in biomedical signal processing and recognition. We first introduce three CNN models for abstracting spatial EEG feature maps. Then, the scheme of the EEG feature fusion for heterogonous modes is presented.

#### 2.5.1. LeNet-5

**Figure 3:**
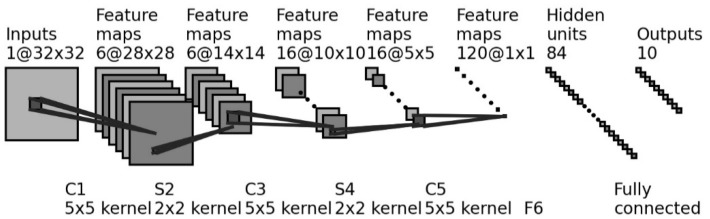
The architecture of LeNet-5.

LeNet-5 is a typical convolutional network proposed by LeCun et al., with eight cascade layers, i.e., an input layer, a convolutional layer (C1), a subsampling layer (S2), a second convolutional layer (C3), a second subsampling layer (S4), a third convolutional layer (C5), a fully connected layer (F6), and an output layer. The architecture of the LeNet-5 is shown in Figure 3. The role of each layer is introduced as follows.

Layer C1 is set up for feature extraction. The input of EEG spatial features is operated with the convolution kernel size of 5 × 5, and then six feature maps are abstracted. Layer S2 is used for data dimension reduction. The size of each feature map is reduced to half by scanning a 2 × 2 patch. In order to reduce the data dimension, the activations of four nodes selected by the 2 × 2 patch are averaged. Its outputs are multiplied by a trainable weight matrix and added with a trainable bias vector. The sigmoid function is used as the activate function.

Layer C3 is similar to C1, while 16 feature maps are abstracted after the convolutional operation with the kernel size of 5 × 5. Similar to S2, S4 has 16 feature maps with the size of 5 × 5. Layer C5 is also a convolutional layer that has 120 feature maps after a convolutional operation. After applying the 5 × 5 convolution kernel on each feature map, the output size of C5 becomes a vector. Layer F6 has 84 units, all of which are fully connected to C5.

#### 2.5.2. GoogLeNet

GoogLeNet is a deeper and wider convolutional network but with less trainable parameters compared to other CNNs with large depth. A large depth of network (22 layers with trainable parameters) and the possession of the Inception module improve the performance of GoogLeNet.

**Figure 4:**
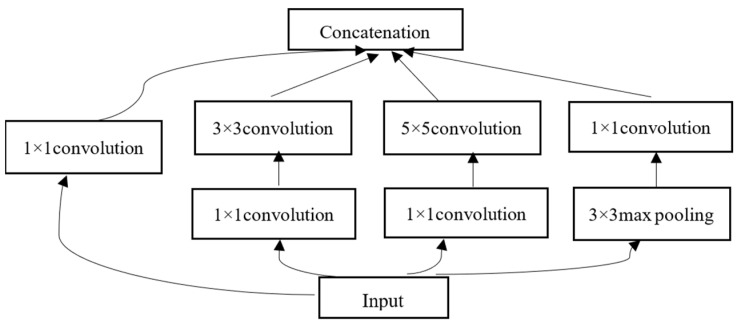
The architecture of the Inception V1 module.

The Inception module aims to achieve a better representation of the input by the use of several convolutional kernels of different sizes to realize perceptions on different scales. The original Inception module consists of four parts: 1 × 1 convolutions, 3 × 3 convolutions, 5 × 5 convolutions, and 3 × 3 max pooling. The input EEG spatial map is sent into four parallel subnetworks. After several convolution and pooling operations, the outputs of each part are concatenated, which form the final output of the Inception module. To reduce the computational cost, GoogLeNet adopts the Inception V1 based on the naïve Inception module. The 1 × 1 convolutions are used to compute reduced feature maps before the subnetworks and a rectified linear activation function is applied as shown in Figure 4.

The structure of GoogLeNet is shown in Table 3. In addition to the Inception module, the convolution layers and pooling layers are similar to popular CNNs like VGG and AlexNet. It is remarkable that GoogLeNet replaces three fully connected layers with an average pool layer and dropout layer to prevent overfitting.

#### 2.5.3. EfficientNet

There are three common approaches to improving the performance of the network: increasing one of network width, depth, or resolution. The CNN model with a larger width of the kernel can capture fine-grain features, but it has difficulties in extracting high-level abstractions. Deeper CNN is able to extract richer features but has difficulty in training due to the vanishing of gradients. Increasing the resolution of the input EEG spatial map will obtain fine-grain patterns at the cost of increased computational time. The EfficientNet is a CNN architecture that balances three schemes to achieve competitive accuracy at a lower computational cost.

Table 4 shows the basic architecture of EfficientNet-B0 used in this study. The MBConv block uses the swish function as the activate function and adds a Squeeze-and-Excitation (SE) module. In an MBConv block, the input is sent into a 1 × 1 convolutional layer to increase the dimension. The number of convolutional kernels is *n* times the input channels. Then, a *k × k* depth-wise convolutional layer is applied. Feature maps obtained by the previous layer are sent into the SE module, which is formed by an average pooling layer and two fully connected layers. The outputs of the SE then go through a 1 × 1 convolutional layer and a dropout layer to find high-level feature representations.

#### 2.5.4. Feature Fusion

Integrating different scales of features is an important method to improve classification performance. Low-level features have higher resolutions, while high-level features have more useful information. How to fuse these EEG feature abstractions in an effective way is the key to improving the generalization capacity of the deep CNN model. In general, feature fusion can be cataloged in two ways by the sequence of fusion and prediction, namely early fusion and late fusion. Here, we employ the scheme of early fusion at the feature level. Principal component analysis (PCA) is used to find the low dimensional embedding of the output abstractions from LeNet5, GoogLeNet, and EfficientNet.

The main purpose of the PCA is to reduce the dimension of features and to discover principal directions embedded in the original feature space. First, each dimension of the feature abstraction is standardized into the values with zero mean and unit variance. Second, assume that a unit vector with maximum variance is mapped from original feature abstractions. The variance can be defined as

(1) $$Var(X_{project})=\frac{1}{m}\sum_{i=1}^{m}{\left\|X_{project}^{\left(i\right)}-{\bar{x}}_{project}\right\|}^{2}.$$

Due to the standardized operation, Euation (1) can be written as

(2) $$Var(X_{project})=\frac{1}{m}\sum_{i=1}^{m}{\left\|X_{project}^{\left(i\right)}\right\|}^{2}.$$

The process of finding the mapping matrix is equivalent to evaluating the maximum of the variance, i.e.,

(3) $$\max\;Var(X_{project})=\frac{1}{m}\sum_{i=1}^{m}{\left(w^{(i)T}\cdot X^{\left(i\right)}\right)}^{2}.$$

Therefore, the output of the PCA model can be computed as follows:

(4) $$\tilde{x}=W^{T}\cdot {\left[x_{L}{}^{T},x_{G}{}^{T},x_{E}{}^{T}\right]}^{T}.$$

In the equation, $x_{L}{}^{T}$, $x_{G}{}^{T}$, and $x_{E}{}^{T}$ are the feature abstractions computed by the LeNet5, GoogLeNet, and EfficientNet, respectively.

#### 2.5.5. Performance Indicators

To evaluate the classification performance of the cognitive workload classifier, indicators including accuracy (ACC), sensitivity (SEN), and specificity (SPE) are used and described as follows.

(5) $$P_{sen}=\frac{TP}{TP+FN}$$

(6) $$P_{spe}=\frac{TN}{TN+FP}$$

(7) $$P_{acc}=\frac{TP+TN}{TP+TN+FP+FN}$$

where *TP* (true positive) indicates that the number of EEG feature vectors corresponding to unambiguous pronoun resolution is correctly classified. The *FP* (false positive) refers to the number of the EEG feature vectors of ambiguous pronoun resolution that are misclassified as unambiguous conditions. The *TN* (true negative) indicates the number of the EEG feature vectors corresponding to the ambiguous pronoun resolution is correctly classified. The *FN* (false negative) indicates misclassification as unambiguous pronoun resolution.

## 3. Results

The results of the cognitive workload assessment experiment include five parts. First, the results of reaction time analysis are presented. Second, the difference of EEG features under two workload states is described. Third, the accuracies of workload classification computed in different combinations of models are reported. Fourth, performance comparisons with different classifiers are illustrated. Fifth, classification accuracies with ensemble models are reported. Data from two participants were excluded due to seriously incomplete records of (i.e., less than 50% of the total) EEG signals. Based on the sampling rate of 128 Hz and the time required for 200 trials (12 s for each trial), the data volume of the two participants among 17 did not conform to the data volume calculated by the experimental recording time. Therefore, all data analyses were based on 15 participants.

### 3.1. Reaction Time Analysis

In Table 5, we list the number and the rate of the 15 participants’ correct answers to the cases of unambiguous pronoun resolution, namely the Type 1 behavioral stimuli. All participants except P8 achieved a score higher than 92%. This suggests that pronouns with a clear gender reference can be accurately identified. It should be noted that the low accuracy (i.e., 0.5625) obtained by P8 may indicate the participant did not correctly understand the instructions of the experiment or did not take the task seriously. The proportion of responses made by the participants to the Type 0 stimuli (that is, the cases of ambiguous pronoun resolution) is reported in Table 6.

**Figure 5:**
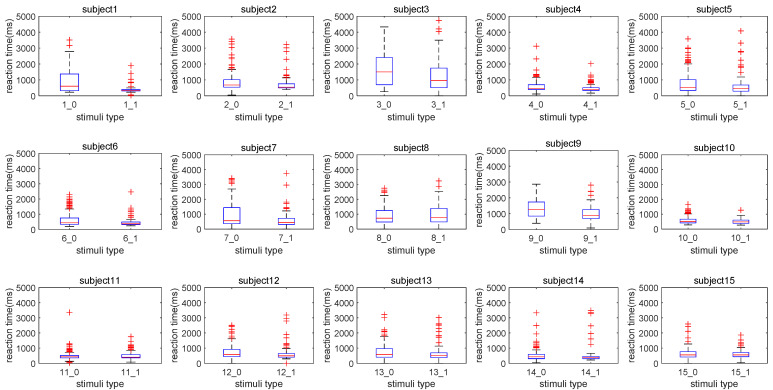
Box plot of reaction times (ms) under two stimuli types for each participant.

We then compared the participants’ reaction times in the cases of ambiguous pronoun resolution (i.e., Type 0) to those in unambiguous conditions (i.e., Type 1). The values of the reaction time are visualized with box plots in Figure 5. We find that most of the participants responded quickly when there was a clear gender reference. However, two types of data from P10, P11, and P15 are comparable in distributions. For P8, the data show an opposite result compared to the others. We interpret the behavioral data of P8 as an anomaly considering his low accuracy rate in the case of Type 1 (see Table 5). A statistical test of the reaction times of 15 participants under different stimuli types was performed via two-sample *t*-tests. For each participant, there were two sets of reaction times: one set contained his reaction times under the case of ambiguous pronoun resolution, and the other set contained his reaction times under unambiguous conditions. We listed the mean and variance of the reaction times for the two types, respectively, and obtained the statistics of reaction times with different sample sizes through a two-sample *t*-test. The corresponding results are shown in Table 7. It can be noted that there are significant differences in reaction times between the two stimuli types for half of the participants, and their mean differences are generally larger than those of the other half. In addition, by comparing the mean differences, it can be found that, except for P11, P14, and P15, all participants have significant mean differences. Combined with the analysis of accuracy rate reported in the case of Type 1 stimuli, in general, we propose that the distribution of reaction time shows both a significance and a mean difference between the data corresponding to Type 1 and Type 0 stimuli, suggesting the rationality of label selection for the EEG data.

### 3.2. Difference of EEG Features under Two Workload States

**Figure 6:**
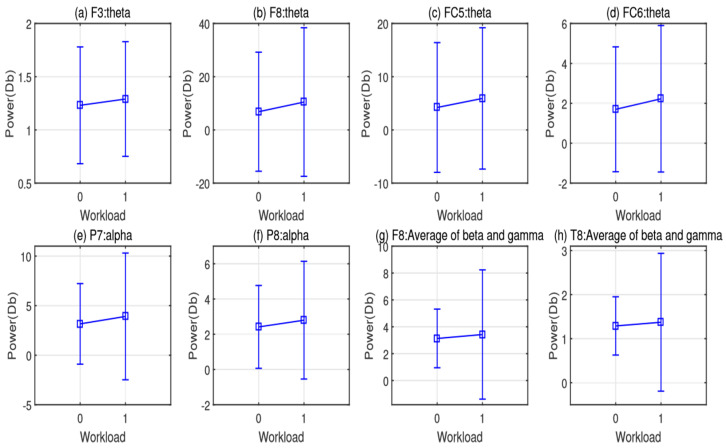
Subfigures (**a**–**h**) denote significantly varied EEG feature distributions under two cognitive states, respectively. Class 0 indicates the cases of ambiguous pronoun resolution. Class 1 indicates the cases of unambiguous conditions, giving the gender information in the context.

To validate whether the mean value of the EEG features between the two cognitive states significantly varied or not, we applied a two-sample *t*-test on all 42 features extracted from the EEG data of all 15 participants. The sizes of the EEG feature samples are 1800 and 1200 under Class 0 and 1, respectively. The corresponding *p* values and *t*-statistics are listed in Table 8. In total, significant variations were observed for eight features, i.e., theta band PSDs of F3, F8, FC5, and FC6 channels; alpha band PSDs of P7 and P8 channels; and the average PSDs of beta and gamma of F8 and T8 channels. The means and corresponding standard deviations of these significant features are plotted in Figure 6.

From Figure 6, it can be seen that all the significant feature values increase from Class 0 to Class 1. Class 0 corresponds to the cases where the pronoun’s referent is undetermined (i.e., Type 0 stimuli), while Class 1 corresponds to the cases of unambiguous conditions (i.e., Type 1 stimuli) providing the gender information of the pronoun and the nouns in context. The increase of the theta band PSD in the frontal cortical region can be thus interpreted as that additional cognitive capacity required to analyze the relationship between implicit gender information and the meaning of the pronoun. On the one hand, the EEG signals collected from electrodes P7 and P8 reflect the neural activations from the bilateral parietal cortex, which is involved in the decision-making process of ambiguous pronoun resolution [16,17]. On the other hand, the EEG feature of power spectral density (PSD) from alpha, beta, and gamma reflects the level of cognitive workload [45]. Accordingly, the variation of the alpha PSD of P7 and P8 and beta and gamma PSD may show cognitive stress induced by the ambiguous pronouns.

**Figure 7:**
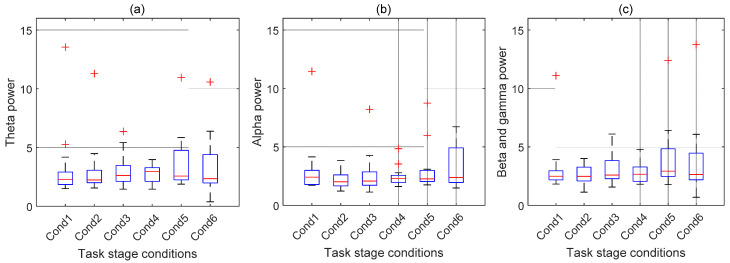
Feature values averaged from all channels in different frequency bands: (**a**) alpha, (**b**) theta, and (**c**) average of beta and gamma are compared across six baseline conditions. Cond 1 denotes the baseline EEG recorded before the experiment of the first trial. Cond 2 to Cond 6 denote the baseline EEG recorded after blocks 1 to 5, respectively. Note that each block contains 40 trials. Some of the outliers are out of scope and not displayed. Each column of data denotes the feature values from 15 subjects.

To avoid the effect of mental habituation on our results of cognitive workload, we analyzed the fatigue level across the whole experimental process of five blocks. In Figure 7, we show the boxplot across six baseline conditions before and after each experimental block to investigate how mental habituation affects the EEG power features. From the figure, we notice that there is no strong linear pattern of the median, which varies from Cond 1 to Cond 6. This implies the power value with theta, alpha, the sum of the beta, and gamma bands does not monotonically increase or decrease along with the time of the experiment. On the other hand, the IQR of each condition seems to increase, and the highest median is seen at Cond 4 or Cond 5. This observation may show the fatigue accumulation under the last several conditions. However, the variations of the means between the last condition and the previous conditions are not significant according to the two-tailed paired *t*-test (Bonferroni correction is performed). Therefore, the observed mental habituation may not significantly affect the results of the cognitive workload classification based on the EEG power feature.

### 3.3. Accuracy of Workload Classification

**Figure 8:**
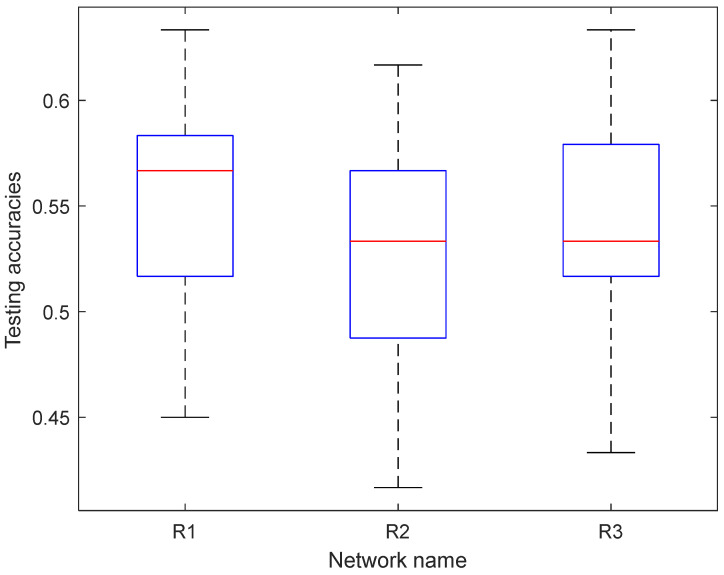
Comparison of the average workload classification accuracy of the three sets of feature representations (denoted as R1 (LeNet5), R2 (GoogleNet), and R3 (EfficientNet)).

To validate the workload classifier performance, we merged all EEG data of 15 participants and used the hold-out method to train a LeNet5 model by setting training and test sets of 70% and 30% of all feature vectors. The original size of each feature map was transformed by an EEG topology graph with the size of 3 × 67 × 67. The three channels are the theta, alpha, and the average of beta and gamma PSDs. The value 67 represents interpolated resolution, which was determined by trial and error. Specifically, the feature map was downsampled to obtain a feature map of 32 × 32 size to accommodate the size of the input layer of the LeNet5. We applied the same structure as the standard LeNet-5 model though updating the input size to 67 × 67 to process more information. In addition, two pre-trained models, GoogLeNet and EfficientNet, were added to use the hold-out method for each subject under the same proportion for training and testing sets. The feature map was upsampled to fit the input size of each network. The feature representations of the training set and test set based on these three networks were obtained and labeled as R1, R2, and R3, for LeNet5, GoogLeNet, and EfficientNet, respectively. The fully connected layer was then added and trained by the target cognitive workload classes. The comparison of the average workload classification accuracy of all 15 participants is shown in Figure 8. The results show that the test accuracy of R3 (0.5544) outperforms R1 and R2. Table 9 shows the performance indicators of testing accuracy, F1-score, SEN, and SPE of the LeNet5 network. Among them, Subject 9 had the highest accuracy (63.33%), and Subject 6 had the lowest accuracy (45.00%). We also recorded the test results of the other two networks in detail. Although the accuracy in some subjects is lower, the accuracies are significantly higher than 50%, based on the results of the *t*-test (*M* = 0.55; *SD* = 0.05; *t* (14) = 4.24; *p* < 0.001).

### 3.4. Performance Comparison with Different Classifiers

In this section, we replace the last fully connected layers with three different shallow learners, i.e., C-support vector machines (C-SVM), naive Bayesian classifier (NB), and k-nearest neighbor (KNN) classifiers, to improve the classification performance. Based on the results shown in Table 10, in the test results of data set R1 among the three learners, the C-SVM combined with the LeNet5 has the highest test accuracy (0.5678). The combination of the LeNet5 and C-SVM also achieves the highest F1-score (0.7018). This implies that the complex models may lead to potential overfitting and impair classification performance.

### 3.5. Classification Accuracy with Ensemble Models

The feature representations of training and testing sets were obtained from the last pooling layer of LeNet5, GoogleNet, and EfficientNet. They were then denoted as R1-TR, R1-TE, R2-TR, R3-TE, R3-TR, and R3-TE, where TR and TE indicate training and testing feature presentations, respectively. In Table 11, the sizes of training and testing sets of each participant are listed. Then, we conducted a study of four cases and recorded the test performance indicators for each case.

Under Case 1, we combined R1-TR and R2-TR of the training set obtained by LeNet-5 and GoogleNet into a new training set (140 × 1144) and combined R1-TE and R2-TE as a new testing set (60 × 1144). The features were then fused with the PCA model. After dimensionality reduction, the size of the training and testing sets were 140 × 139 and *t* 60 × 139, respectively.

Under Case 2, we combined the feature representations obtained by LeNet-5 and EfficientNet to build a new training set (140 × 1400) and a new testing set (60 × 1400). Based on the PCA processing, the dimensions of the training and testing sets were reduced to 139.

**Figure 9:**
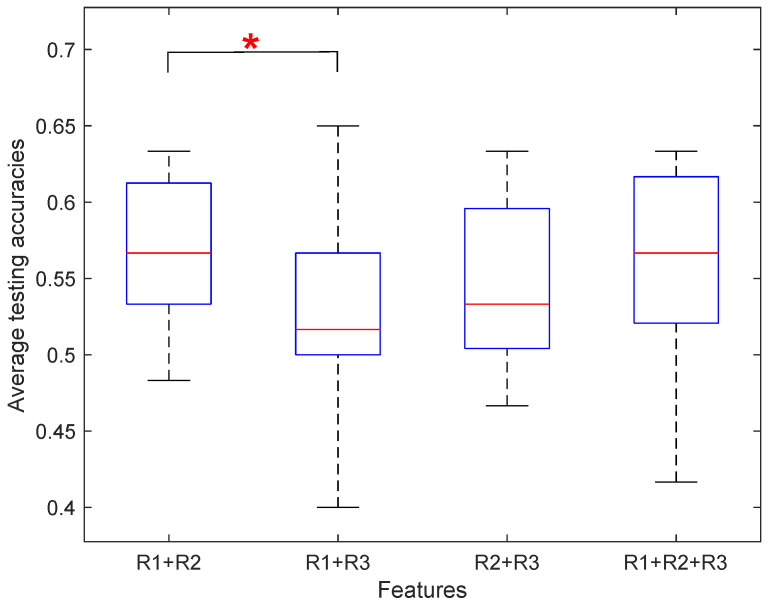
Average testing accuracies of the four cases with R1: Lenet-5, R2: GoogleNet, R3: EfficientNet. Accuracy differing from the baseline case of R1 + R2 is indicated with an asterisk (* *p* < 0.05).

Under Case 3, we combined the features obtained by GoogleNet and EfficientNet into a new training set (140 × 2304) and a new testing set (60 × 2304). Under Case 4, we combined all feature representations obtained by Lenet-5, GoogleNet, and EfficientNet into a new training set (140 × 2314) and a new testing set (60 × 2314). Table 12 shows the leave-out cross-validation test results for Case 1, including ACC, F1-score, SEN, and SPE. Subject 15 had the highest accuracy (63.33%), and Subject 9 had the lowest accuracy (48.33%). The overall accuracies are significantly higher than 50%, based on the results of the *t*-test (*M* = 0.58; SD = 0.04; *t* (14) = 5.85; *p* < 0.0001). Table 13 shows the 10-fold cross-validation test results for the fusion of LeNet-5 and GoogleNet. Subjects 2 and 13 had the highest accuracy (62.00%), and Subject 9 had the lowest accuracy (46.50%). Although the accuracy in some subjects is lower, the overall accuracies are significantly higher than 50%, based on the results of the *t*-test (*M* = 0.56; SD = 0.05; *t* (14) = 4.69; *p* < 0.001). The results of the other three cases were also recorded in detail. In Figure 9, the average accuracy of the four cases is shown. Figure 9 illustrates that the feature combination of LeNet5 and GoogLeNet possesses the best performance, which is slightly higher than the case when the single model was applied. This performance is comparable with the case where LeNet5 and C-SVM are combined to predict the workload.

## 4. Discussion

According to the analysis of participants’ performances on the pronoun resolution trials, their reaction times, and the EEG distributions, we discover that the change of the manipulated task condition can induce the variation of the external behavior indicator of the main task. When gender information was not sufficient to identify the referent of a pronoun in the Chinese context, we observed a relatively longer reaction time consumed by pronoun resolution compared to unambiguous controls (Figure 5 and Table 7). In contrast, our EEG results suggested that unambiguous pronoun resolution was associated with a heavier cognitive workload than its ambiguous counterpart (Table 8 and Figure 6). We hypothesize that different cognitive mechanisms were recruited during the resolution procedures of different referential types, namely ambiguous and unambiguous pronouns. We argue that the cognitive workload as an indicator at a neurophysiological level may provide complementary information to understand the cognitive mechanisms of pronoun resolution. Specifically, we discuss the role of the behavioral representations and the cognitive workload in discovering the cognitive procedures of pronoun resolution based on a tripartite structure of analysis at different levels, namely the behavioral, neural, and algorithm levels.

To understand the cognitive mechanisms underlying pronoun resolution, we first compare our experimental results at the behavioral level. The overall high accuracy rate (Table 5) in unambiguous conditions suggests that gender information could be used to determine the referent of an unambiguous pronoun and that all participants except one (i.e., P8) actively participated in our cognitive task of pronoun resolution. Previous investigations have reported the usefulness of gender information in determining a correct referent [4,16,17]. We observed the consistency of the participants’ selections (Table 6) in the ambiguous cases, which have previously been reported to reflect the fact that other linguistic cues such as verb bias could be used in identifying a pronoun’s referent [8]. The reaction times, on the whole, revealed that ambiguous pronoun resolution was correlated to a more time-consuming cognitive mechanism compared to unambiguous controls. Several investigations also have implicated longer reading time for referentially ambiguous words [54,55]. For seven participants in our experiments, namely P1, P3, P4, P6, P7, P9, and P10, the significantly increased reaction time suggested an increased processing difficulty when ambiguous pronouns were identified by operators. Based on our literature review, we hypothesize that a two-step mechanism of decision making contributes to ambiguous pronoun resolution compared to unambiguous controls, causing greater reaction time.

At the neural level, our EEG signals suggested a difference in cognitive workload recruited in ambiguous pronoun resolution in comparison to unambiguous conditions. For all participants, we extracted the EEG power features of multiple frequency bands. For each feature vector, we computed the power of theta (4–8 Hz), alpha (8–13 Hz), and the mean power of beta (14–30 Hz) and gamma (31–40 Hz). Results of our analysis on the mean values of the EEG features of these frequencies suggest significant differences between the two cognitive states underlying ambiguous pronoun resolution compared to the unambiguous controls. Specifically, we observed significant differences in the bilateral frontal area (F3, F8, FC5, FC6 channels) from theta, beta, and gamma, and in the bilateral parietal area (P7, P8) from theta (Table 8 and Figure 6). The theta power across two task conditions decreased and thus suggested a lower cognitive workload level correlated to a shorter reaction time. This was supported by previous studies of cognitive workload [41,44,49]. Such a contradiction could be interpreted as the cognitive capacity needed to identify a pronoun’s referent determined by the gender information. In other words, a greater cognitive workload was required when a participant realized that the cognitive task had a standard answer in the cases of unambiguous pronoun resolution. On the contrary, when the pronoun’s referent was undetermined, the participant might feel freer to complete the resolution task in a more flexible way, processing the information given in the context. Such a cognitive process may not leverage additional working memory resources.

At the level of algorithm, we evaluated different computational models in the domain of machine learning methods. To differentiate between the neural states recruited in the two types of pronoun resolutions, we transformed the EEG feature map into an optimized EEG topology graph, aiming at validating the workload classifier performance. The original size of each feature map was transformed into an EEG topology graph with the size of 3 × 67 × 67, where 3 represents three features of the band frequencies, and 67 represents the interpolated resolution determined by trial and error. Based on this EEG topology, several computational models were evaluated in the domain of machine learning. To explore which network used in the research had relatively excellent performance, the results obtained from the experiments were analyzed. It could be seen from the results that EfficientNet performed the best of the datasets, with the highest accuracy of 55.44% (Figure 8). These results were basically in line with previous findings on the accuracy of these models. EfficientNet has a deeper and wider architecture in contrast with LeNet-5 and GoogLeNet [52]. A more complex architecture might lead to better performance by obtaining features with more useful information. In a seminal study, several different classifiers were compared, and the Support Vector Machine (SVM) was reported to obtain the highest accuracies among other popular classifiers, such as K-Nearest Neighbor (KNN) [34]. In the current study, after a close examination of different classifiers, C-SVM stood out, with the highest accuracy of 56.78% (Table 10). It could be inferred that C-SVM was more suitable than other classifiers when it came to the identification of cognitive workload level. Four combined models were put forward to test the performance. According to the experimental results, the feature combination of LeNet5 and GoogLeNet possesses the best performance on the dataset, with an accuracy of 56.67% with a leave-one-subject-out cross-validation test (Table 12 and Figure 9). Mean accuracy obtained by the same combination was reported as 55.73% with a ten-fold cross-validation test (Table 13). Although EfficientNet had a good performance as a single model, the accuracy was not improved when it was combined with the other two networks. This is probably due to the differences in network structures.

Our investigation had the motivation of studying the difference in the responses to ambiguous pronouns compared to unambiguous controls in the perspective of cognitive workload. We collected not only behavioral data, including reaction time and response selection, but also the EEG signals during the pronoun resolution task. Together, these results highlight cognitive workload as a complementary indicator of differentiating between the cognitive procedures underlying ambiguous and unambiguous pronoun resolution. The other contribution of the present study is to apply pre-trained deep convolutional neural networks (CNNs) to represent spatial patterns of the EEG power features of multiple frequency bands. It is worth noting that the moderate accuracies that we gained will not affect any of our main conclusions. High accuracies may be considered a necessary criterion in studies of emotion recognition or fatigue detection, since human–computer interaction (HCI) systems are expected to identify human emotional states or fatigue levels based on these classification outcomes. In contrast, our study focuses on the cognitive mechanism of human language processing, which involves a more complicated cognitive function than emotion or attention. We collected and analyzed not only EEG data but also behavioral data such as reaction time and participant response. Accordingly, we adopted a tripartite structure of analysis at different levels, namely the behavioral, neural, and algorithm levels. Classification accuracy contributes only one part of our outcome, at the algorithm level. Indeed, we expect a multi-level assessment of the cognitive mechanisms, rather than an application of the accuracies in the identification of ambiguous/unambiguous pronoun resolution by an HCI system. This study has limitations. First, linguistic cues other than gender information were not considered during pronoun resolution. Second, the proposed model cannot effectively deal with the individual difference induced by the varied EEG feature distributions across all 15 participants. Third, considering the specific structural characteristics of Chinese, a generalization of the conclusions of the current study will require further research in other languages.

## 5. Conclusions

In this paper, we evaluated variations of cognitive workload under two human–machine interaction conditions in the Chinese context: ambiguous and unambiguous pronoun resolution. In ambiguous conditions compared to unambiguous controls, we observed a contradiction between an increase in reaction time and a decrease of the theta power that corresponded to an increase in the cognitive workload, suggesting that additional working memory resources were occupied when a participant realized that the cognitive task had a standard answer in the cases of unambiguous pronoun resolution. The power features presented in spatial feature maps could be extracted to find high-level abstractions to model the workload. Overall, the workload classification rate by fusing the GoogLeNet and EfficentNet outputs was 55–63% in a participant-specific manner.

While previous studies have suggested that a decision-making procedure may be involved in ambiguous pronoun resolution [17], the present investigation attempted to identify the specific cognitive mechanisms implicated in different types of pronoun resolution. We argue that a more time-consuming mechanism of decision making was recruited in ambiguous pronoun resolution, whereas less cognitive workload was required since the participant could perform with more flexibility. Our work may constitute a promising starting point for multiple levels of research, including not only the investigation of pronoun resolution and its neural basis but also the development of big EEG data analytics based on deep learning. The potential applications of our study in practical concerns may lie in two aspects: diagnosis of dyslexia and the related educational remediation. Defects in pronoun resolution may lead to language misunderstanding and even reading disability, i.e., dyslexia. The current study provides a new perspective to explore the mechanism underlying pronoun resolution by means of cognitive workload assessment and thus will hopefully inspire dyslexia diagnosis and related educational remediation by taking the patients’ level of cognitive workload into consideration. Future research that addresses the cognitive workload brought by language processing will elucidate our overall understanding of the neural network of pronoun resolution. On the practical side, taking into account additional linguistic cues that are related to pronoun resolution can become prohibitive. Further development of algorithms to implement analysis of EEG data in human–machine interactions is needed.

## Figures and Tables

**Table 1:** Rules of gender rating.

Gender of the Noun	Examples	Five-Point Scale	Three-Point Scale
Definitely female	*taitai* ‘madam’	1	3
Weakly female	*hushi* ‘nurse’	2	2
Neutral	*tongshi* ‘colleague’	3	1
Weakly male	*fanren* ‘prisoner’	4	2
Definitely male	*xiaonanhai* ‘little boy’	5	3

**Table 2:** Description of the behavioral stimuli.

Stimuli Type	Pronoun Type	Syntactic Structure	Example
type 0	ambiguous	S-V-O	*Muqin weixie nüer*. ‘The mother threatened the daughter.’
			*Ta shengqi-le.* ‘She was angry.’
		N1-N2-V	*Qizi he xiaonühai jianmian.* ‘The wife met the little girl.’
			*Ta manzu-le.* ‘She satisfied.’
type 1	unambiguous	S-V-O	*Nanren xiangxin nüer.* ‘The man believed the daughter.’
			*Ta xiao-le.* ‘He smiled.’
		N1-N2-V	*Qizi he zhangfu zhengchao.* ‘The wife quarreled with the husband.’
			*Ta shengqi-le*. ‘She was angry.’

S-V-O: subject-verb-object. N1-N2-V: noun-noun-verb.

**Table 3:** The structure of GoogLeNet.

Type	Kernel Size/Stride	Depth	Output Size
Convolution	7 × 7/2	1	112 × 112 × 64
Max pool	3 × 3/2	0	56 × 56 × 64
Convolution	3 × 3/1	2	56 × 56 × 192
Max pool	3 × 3/2	0	28 × 28 × 192
Inception(3a)		2	28 × 28 × 256
Inception(3b)		2	28 × 28 × 480
Max pool	3 × 3/2	0	14 × 14 × 480
Inception(4a)		2	14 × 14 × 512
Inception(4b)		2	14 × 14 × 512
Inception(4c)		2	14 × 14 × 512
Inception(4d)		2	14 × 14 × 528
Inception(4e)		2	14 × 14 × 832
Max pool	3 × 3/2	0	7 × 7 × 832
Inception(5a)		2	7 × 7 × 832
Inception(5b)		2	7 × 7 × 1024
Avg pool	7 × 7/1	0	1 × 1 × 1024
Dropout(40%)		0	1 × 1 × 1024
Linear		1	1 × 1 × 1000
Softmax		0	1 × 1 × 1000

**Table 4:** The architecture of EfficentNet-B0.

Stage	Operator	Resolution	Channel	Layer
1	Conv3 × 3	224 × 224	32	1
2	MBConv1, k3 × 3	112 × 112	16	1
3	MBConv6, k3 × 3	112 × 112	24	2
4	MBConv6, k5 × 5	56 × 56	40	2
5	MBConv6, k3 × 3	28 × 28	80	3
6	MBConv6, k5 × 5	14 × 14	112	3
7	MBConv6, k5 × 5	14 × 14	192	4
8	MBConv6, k3 × 3	7 × 7	320	1
9	Conv1 × 1&Pooling&FC	7 × 7	1280	1

**Table 5:** Number and rate of each participant’s correct answers to Type 1 stimuli.

Participant Index	Number of Correct Answers	Accuracy Rate
#1	78	0.9750
#2	77	0.9625
#3	77	0.9625
#4	78	0.9750
#5	75	0.9375
#6	79	0.9875
#7	75	0.9375
#8	45	0.5625
#9	80	1.0000
#10	79	0.9875
#11	74	0.9250
#12	77	0.9625
#13	74	0.9250
#14	77	0.9625
#15	78	0.9750

**Table 6:** The proportion of responses made by the participants to Type 0 stimuli.

Participant Index	The Proportion of Z	The Proportion of M	The Proportion of None
#1	0.4333	0.5667	0
#2	0.4083	0.5917	0
#3	0.3583	0.6417	0
#4	0.3333	0.6667	0
#5	0.2917	0.6667	0.0417
#6	0.4250	0.5750	0
#7	0.1417	0.8167	0.0417
#8	0.4917	0.4500	0.0583
#9	0.3750	0.6250	0
#10	0.4083	0.5917	0
#11	0.5000	0.5000	0
#12	0.3750	0.6083	0.0167
#13	0.3083	0.6417	0.0500
#14	0.3000	0.7000	0
#15	0.4667	0.5250	0.0083

**Table 7:** Results of the two-sample *t*-test between the reaction times (ms) under two stimuli types for each participant.

Participant Index	*M*0	*V*0(×10^5^)	*M*1	*V*1(×10^5^)	*p*
#1	922.8000	5.5430	394.8250	0.6054	**<0.0001**
#2	906.8083	3.9771	750.3625	3.0268	0.0723
#3	1671.3250	12.6708	1255.1625	9.6150	**0.0077**
#4	600.6000	1.5579	498.1500	0.8538	**0.0484**
#5	817.0417	5.1775	691.6750	5.3187	0.2313
#6	663.7000	2.4416	483.1625	1.0309	**0.0043**
#7	954.4167	6.6568	634.8375	3.4308	**0.0028**
#8	952.7917	3.4874	1042.3250	4.1254	0.3118
#9	1352.3917	3.2787	1024.6000	2.5207	**<0.0001**
#10	585.9167	0.6708	508.0875	0.2722	**0.0181**
#11	489.8833	1.1614	501.4000	0.8066	0.8030
#12	776.2667	2.6158	665.5500	2.9460	0.1449
#13	795.8667	2.9384	716.9125	3.7115	0.3382
#14	556.5417	2.1436	555.7375	4.3380	0.9919
#15	648.1417	1.5386	611.2625	0.8704	0.4746

*M*0: mean reaction time under stimuli of Type 0. *V*0: variance of reaction time under stimuli of Type 0. *M*1: mean reaction time under stimuli of Type 1. *V*1: variance of reaction time under stimuli of Type 1. If the *p* values are less than 0.05, the corresponding value is shown in bold.

**Table 8:** The two-sample *t*-test between the EEG feature values under two cognitive states. The results of three features, i.e., PSDs of the theta, alpha, and average of the beta and gamma bands from 14 EEG channels are shown.

Channel	Theta		Alpha		(Beta + Gamma)/2	
	*p* Value	*T* Value	*p* Value	*T* Value	*p* Value	*T* Value
AF3	0.5606	−0.5820	0.5729	−0.5638	0.3213	−0.9921
AF4	**0.2631**	**−1.1194**	0.6316	−0.4795	0.8329	−0.2109
F3	0.0036	−2.9111	0.4954	−0.6818	0.1418	−1.4696
F4	0.8912	0.1368	0.0707	1.8083	0.0829	−1.7347
P7	0.6886	−0.4008	**<0.0001**	**−3.9306**	0.4052	−0.8325
P8	0.5807	−0.5524	**0.0003**	**−3.6612**	0.2001	−1.2815
O1	0.2673	−1.1096	0.2236	−1.2172	0.1417	−1.4699
O2	0.1478	−1.4479	0.2272	−1.2078	0.6872	−0.4027
F7	0.4698	−0.7229	0.0717	−1.8019	0.4439	−0.7657
F8	**<0.0001**	**−3.9354**	0.9440	−0.0702	**0.0238**	**−2.2619**
FC5	**0.0003**	**−3.6126**	0.3226	−0.9894	0.1056	−1.6187
FC6	**<0.0001**	**−4.2182**	0.2153	−1.2395	0.1850	−1.3260
T7	0.5987	−0.5263	0.2464	1.1595	0.0885	−1.7041
T8	0.5893	−0.5399	0.2876	−1.0635	**0.0443**	**−2.0122**

The significant *p* values are marked in bold.

**Table 9:** Testing accuracy, F1-score, SEN, and SPE of all participants of the LeNet5 network.

Participant Index	*Pacc*	F1-Score	*Psen*	*Pspe*
#1	0.5667	0.7111	0.5926	0.3333
#2	0.5167	0.6667	0.5686	0.2222
#3	0.5333	0.6818	0.5769	0.2500
#4	0.5667	0.7174	0.5893	0.2500
#5	0.5167	0.6588	0.5714	0.2727
#6	0.4500	0.5479	0.5405	0.3043
#7	0.5167	0.6420	0.5778	0.3333
#8	0.6167	0.6761	0.6857	0.5200
#9	0.6333	0.7609	0.6250	0.7500
#10	0.5833	0.7191	0.6038	0.4286
#11	0.5500	0.7097	0.5789	0.0000
#12	0.5667	0.7111	0.5926	0.3333
#13	0.5833	0.7191	0.6038	0.4286
#14	0.6167	0.7356	0.6275	0.5556
#15	0.5000	0.6512	0.5600	0.2000
Mean	0.5544	0.6872	0.5930	0.3455

*Pacc*: participant accuracy. *Psen*: participant sensitivity. *Pspe*: participant specificity.

**Table 10:** Testing accuracy, F1-score, SEN, and SPE of workload classifiers of combined NB, C-SVM, and KNN classifiers with different deep learning models.

Data	*Pacc*	F1-Score	*Psen*	*Pspe*
LeNet5-NB	0.4978	0.4684	0.5862	0.4258
GoogleNet-NB	0.5311	0.5851	0.6187	0.4246
EfficientNet-NB	0.5189	0.5414	0.6064	0.4367
LeNet5-C-SVM	0.5678	0.7018	0.6000	0.3981
GoogleNet-C-SVM	0.5400	0.6129	0.6172	0.4254
EfficientNet-C-SVM	0.5300	0.6014	0.6124	0.4138
LeNet5-KNN	0.5367	0.6352	0.5967	0.4126
GoogleNet-KNN	0.5544	0.6463	0.6189	0.4238
EfficientNet-KNN	0.5022	0.5950	0.5772	0.3726

NB: naive Bayesian classifier. C-SVM: C-support vector machines. KNN: k-nearest neighbor. *Pacc*: participant accuracy. *Psen*: participant sensitivity. *Pspe*: participant specificity.

**Table 11:** The size of the training set and test set in different networks.

Lenet-5		GoogleNet		EfficientNet	
R1-TR	R1-TE	R2-TR	R2-TE	R3-TR	R3-TE
140 × 120	60 × 120	140 × 1024	60 × 1024	140 × 1280	60 × 1280

R1-TR: the training set of LeNet5. R1-TE: the testing set of LeNet5. R2-TR: the training set of GoogleNet. R2-TE: the testing set of GoogleNet. R3-TR: the training set of EfficientNet. R3-TE: the testing set of EfficientNet.

**Table 12:** Leave-out cross-validation test performance indicators of Case 1. The feature representations are fused by LeNet5 and GoogLeNet.

Participant Index	*Pacc*	F1-Score	*Psen*	*Pspe*
#1	0.6167	0.5978	0.6944	0.5000
#2	0.6167	0.5632	0.8056	0.3333
#3	0.5167	0.4745	0.6667	0.2917
#4	0.5333	0.4976	0.6667	0.3333
#5	0.5667	0.5335	0.6944	0.3750
#6	0.5333	0.4750	0.7222	0.2500
#7	0.5333	0.4750	0.7222	0.2500
#8	0.5667	0.5335	0.6944	0.3750
#9	0.4833	0.4489	0.6111	0.2917
#10	0.5667	0.5238	0.7222	0.3333
#11	0.6000	0.5604	0.7500	0.3750
#12	0.5333	0.4444	0.7778	0.1667
#13	0.6167	0.5632	0.8056	0.3333
#14	0.5833	0.5369	0.7500	0.3333
#15	0.6333	0.5875	0.8056	0.3750
Mean	0.5667	0.5210	0.7259	0.3278

*Pacc*: participant accuracy. *Psen*: participant sensitivity. *Pspe*: participant specificity.

**Table 13:** Ten-fold cross-validation test performance indicators of Case 1. The feature representations are fused by LeNet5 and GoogLeNet.

Participant Index	*Pacc*	F1-Score	*Psen*	*Pspe*
#1	0.5500	0.4980	0.7149	0.3254
#2	0.6200	0.5520	0.7984	0.3473
#3	0.5550	0.4826	0.7469	0.2637
#4	0.6000	0.5627	0.7285	0.4485
#5	0.5050	0.4340	0.6663	0.2387
#6	0.6150	0.5739	0.7109	0.4549
#7	0.5350	0.4907	0.6486	0.3477
#8	0.5750	0.5221	0.7148	0.3435
#9	0.4650	0.4138	0.6105	0.2420
#10	0.5150	0.4675	0.6625	0.3103
#11	0.5650	0.5206	0.6899	0.3654
#12	0.4950	0.4512	0.6457	0.2854
#13	0.6200	0.5536	0.7933	0.3608
#14	0.5750	0.5217	0.7404	0.3936
#15	0.5700	0.5278	0.7172	0.3776
Mean	0.5573	0.5048	0.7059	0.3403

*Pacc*: participant accuracy. *Psen*: participant sensitivity. *Pspe*: participant specificity.

## Data Availability

We choose to exclude this statement.
